# Electroosmotically generated disinfectant from urine as a by-product of electricity in microbial fuel cell for the inactivation of pathogenic species

**DOI:** 10.1038/s41598-020-60626-x

**Published:** 2020-03-26

**Authors:** Iwona Gajda, Oluwatosin Obata, John Greenman, Ioannis A. Ieropoulos

**Affiliations:** 1 Bristol BioEnergy Centre, Bristol Robotics Laboratory, University of the West of England, Bristol, BS16 1QY UK; 2 Biological, Biomedical and Analytical Sciences, University of the West of England, Bristol, BS16 1QY UK

**Keywords:** Microbiology, Environmental sciences, Energy science and technology

## Abstract

This work presents a small scale and low cost ceramic based microbial fuel cell, utilising human urine into electricity, while producing clean catholyte into an initially empty cathode chamber through the process of electro-osmostic drag. It is the first time that the catholyte obtained as a by-product of electricity generation from urine was transparent in colour and reached pH>13 with high ionic conductivity values. The catholyte was collected and used *ex situ* as a killing agent for the inactivation of a pathogenic species such as *Salmonella typhimurium*, using a luminometer assay. Results showed that the catholyte solutions were efficacious in the inactivation of the pathogen organism even when diluted up to 1:10, resulting in more than 5 log-fold reduction in 4 min. Long-term impact of the catholyte on the pathogen killing was evaluated by plating *Salmonella typhimurium* on agar plates and showed that the catholyte possesses a long-term killing efficacy and continued to inhibit pathogen growth for 10 days.

## Introduction

Addressing waste management and global climate change issues effectively is important for the sustainable development and circular economy, where waste products should be reused and recycled. In recent years microbial bioelectrochemical technologies are being explored for value-added resource recovery in the form of interaction between microorganisms and electrodes^1^. One of the technologies is a microbial fuel cell (MFC), which produces direct electrical current from the anaerobic oxidation of biodegradable organic waste^2,3^. The biofilm anode of the MFC is the electron acceptor for anode-respiring bacteria, which liberates electrons from organic compounds and transports them to a cathode, where we can harvest valuable products^4^. Wastewater is a source of nutrients and source separated urine, due to high concentration of ions, can be considered as a valuable resource for N, P, K^5,6^, sensing^7^ and energy recovery^8^, where urine can serve as a source of hydrogen^9,10^ or direct electricity via MFC^8^ to power LED lighting as a demonstration of scaled-up systems^11^. Separated urine as a concentrated stream could make wastewater management easier becoming a source of electrical current, nutrients for fertilizers and disinfectants. This could be achieved within a MFC system, which is purposefully designed to achieve good levels of power performance and – at the same time separate urine feed into the anolyte and newly formed filtrate (catholyte) forming in the cathode chamber^12^. During the MFC operation, as the protons and cations migrate from the anode through the membrane to the cathode, water molecules are simultaneously moved by electro-osmotic drag^13,14^ resulting in accumulation of liquid in air-breathing cathode that maintains hydration and prevents salt accumulation both in wastewater^15,16^ and urine operated MFCs^12,17^. In urine-fed MFCs, the majority of transported ions are NH_4_^+^^18^ that act as proton shuttles^19^ resulting in ammonia removal from the urine stream and its recovery in the cathodic part of the system.

The product of cathodic Oxygen Reduction Reaction (ORR) is OH- whose accumulation results in cathodic pH increase. This can be harvested as a caustic agent^20^ that potentially could serve as a disinfectant deactivating *E.coli* in previously shown wastewater operated MFCs^21^. Catholyte production through electroosmosis and cathodic ORR activity is a particularily useful feature to obtain power output and simultaneous production of extracted caustic agent in the form of catholyte (as a byproduct) that could be recovered from the system and used as a bactericide especially in remote locations and decentralised systems or as an alternative to environment-polluting chemical disinfectants.

Within the broad spectrum of current disinfection technologies, electrolysis has emerged as a very interesting alternative, for the production of hydrogen peroxide^22^ or electro-generated Reactive Oxygen Species (ROS)^23^ through the electro-Fenton process^24^. This is with a supplied energy source to drive electrolysis and in the case of the Fenton reaction, an addition of ferric ion. However, disinfectants produced in the electrochemical cell with no addition of external energy or chemicals are desirable to make the system more sustainable.

This work presents a low cost ceramic based MFC with a built-in inner cathode chamber for catholyte production and accumulation that is generating electrical current and simultaneously producing highly disinfecting catholyte *in situ* only when the electricity is produced. The bacterial deactivation is studied here using kill curves on *Salmonella typhimurium* using neat and diluted catholyte. This is the first time that the production of transparent catholyte of disinfecting properties is produced from urine operated MFC.

## Materials and Methods

### MFC configuration and operation mode

**Figure 1:**
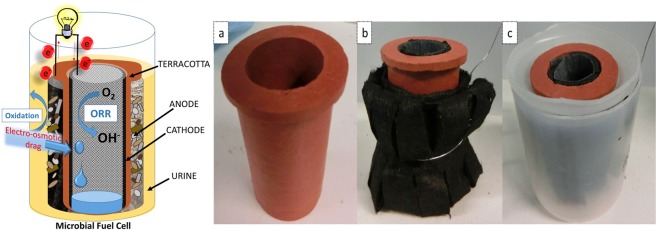
Ceramic MFC chassis (**a**), MFC with an outer anode and inner cathode (**b**) and the MFC assembled within the anodic chamber (**c**).

Single-chamber air-breathing cathode microbial fuel cells were used in these experiments where the ceramic structure acted as the separator between the anode and the cathode. Ceramic cylinders were made from terracotta clay (India), 5 cm tall with a wall thickness of 3 mm and the inner diameter of 2.2 cm. The top of the cylinder had a lip in order to secure the cylinder in position within the outer anodic container (Fig. 1). Porosity of the ceramic membrane was measured by the water absorption method and showed that the ceramic used in this study had an open porosity of 12.3 %.

Anode materials preparation: All anodes were prepared on the basis of the identical 30 × 20 cm cut pieces of 20 g/m^2^ carbon veil fibre (PRF Composites, UK) where carbon veil was coated with activated microporous ink. The ink was prepared by blending 40 g of activated carbon (G Baldwins and Co.) with 5% PTFE (60% water dispersion in H2O, Sigma Aldrich) and 300 mL of deionised water. The mixture was stirred for 2 minutes -to obtain a slurry and applied onto both sides of carbon veil. The sheets of such prepared material were then heat treated in an oven at 250 ° C for 30 min. This modification was performed with the activated carbon loading of 5 mg/cm^2^. The total carbon loading of the anodic material was 25 g/m^2^.

The cathodes were prepared with activated carbon powder mixed with PTFE and applied on PTFE treated carbon veil as previously described^25^.

The air-cathode (22.5 cm^2^) was placed inside the cylinder where the activated carbon was in direct contact with the ceramic surface (Fig. 1.). Plastic mesh rollers were placed inside the cylinders to maintain stable contact between the cathode and the terracotta as well as to obtain good visibility into the cathode chamber that is aimed to collect extracted catholyte. Stainless steel crocodile clips were used to connect to the cathodes and cathode chambers were covered with parafilm to restrict evaporation. The plastic cylindircal anodic containers were made of plastic bottles with inlets and outlets and sealed with non-toxic sealant (Wet Water Sticky Stuff, UK) in order to connect to the continuous feeding and peristaltic pump (16-channel Watson Marlow 205, Falmouth, UK).

MFC reactors were inoculated using 72 mL of inoculum solution that consisted of activated anaerobic sludge collected from Wessex Water treatment plant (Saltford, UK) mixed with hydrolysed (24 h old, pH 9–9.2) human urine in the ratio of 1:1. Human urine was collected anonymously from consented, healthy adults with no known previous medical conditions and pooled together in a 40 L tank. Undiluted urine was then collected from the same tank and fed directly from the 5L reservoir into the MFCs in continuous flow using the multi-channel peristaltic pump at a flow rate of 242 mL/day. All experimental methods were carried out in accordance with relevant guidelines and regulations and were approved by the Faculty of Engineering and Technology Research Ethics Committee (FREC) at the University of the West of England, number 12/YH/0493.

### Analysis and calculation

The output voltage was measured across an external resistor (100 Ω) using an Agilent 34970 A data acquisition system connected to a PC; the 100 Ω resistor was selected on the basis of previous experiments with identical MFCs, following polarisation runs. To electrochemically characterise the anode and cathode half-cells, linear sweep voltammetry (LSV) was performed using an electrochemical workstation (SP-50, Bio-Logic), with a three-electrode configuration consisting of working electrode (tested MFC electrode), a Ag/AgCl reference electrode and a counter electrode (MFC counter electrode for given test) of the tested MFCs. Polarisation curves of the matured MFCs were performed in a two-electrode configuration with the working channel connected to the anode and the counter channel was short circuited with the reference channel and connected to the cathode. The scan rate was 0.25 mV.s^−1^ from OCV to 0.02 V.

### Catholyte efficacy as a bactericidal agent

To evaluate the killing properties of catholyte derived from current generating MFCs, bioluminescent *Salmonella typhimurium* (10^8^) was employed. This strain carries the pBBR1MCS-2 plasmid derivative containing the luxCDABE operon of *Photorhabdus luminescens* allowing real time assessment of the impacts of killing agents. The bacteria were grown on nutrient agar overnight, transferred to 50 mL nutrient broth and incubated at 37 °C overnight, resulting in a biomass density of 1 × 10^8^. Subsequently, 0.5 mL of bacterial culture was added to a clean 1.5 mL Eppendorf tube (Fisher Scientific, Loughborough, United Kingdom) for bioluminescence measurement using a single-tube FB12 luminometer (Berthold Detection Systems, Germany). Neat catholyte (~pH 13.4) was used as the test killing agent while 1 M NaOH (pH 13.4) was employed at the positive control. To evaluate the killing efficacy of the killing agents, 0.5 mL of the different catholyte solutions and NaOH was added to the 0.5 mL of pathogenic organism in the 1.5 Eppendorff tube and relative light unit measurements were recorded. The automated protocol included a 3 s delay to allow for the reading of measurements. Bacterial bioluminescence was recorded every 15 s for the first minute and then at 60 s intervals. Relative light emission (RLU) was plotted to show kill kinetics of the pathogenic organism as previously described^21^. To evaluate the impact of dilutions on the efficacy of the killing agents, the catholyte and NaOH were diluted with deionised water (pH 6.9) in 1:1, 1:5 and 1:10. Same volume of the diluted solutions were added to the target organisms as stated above. To evaluate the impact of the pH of the killing agents on their efficacy, droplets of 1 M H_2_SO_4_ were added to the different catholyte solutions and NaOH to obtain pH 7.2 and 9.3. 50 µL of the killing agents were added to inoculated agar plates containing *Salmonella typhimurium* (10^8^/mL) incubated at 37 ^o^C and observed for 10 days. This was to evaluate the long-term efficacy of the killing agent and to determine if the killing activities are reversible or if there is loss of efficacy with time.

### Statistical analysis

Statistical analyses were performed using GraphPad Prism (GraphPad Software, San Diego, CA, USA). Statistical significance was determined using 2-way analysis of variance (ANOVA) with 95% confidence interval (CI).

## Results

### Initial power performance and catholyte production

**Figure 2:**
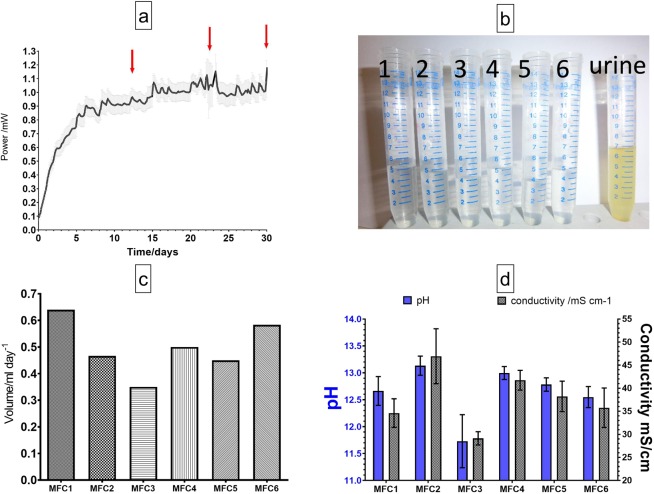
First 30 days of operation of all six MFCs; red arrows show catholyte collection points (**a**), photograph of the catholyte produced from all tested MFCs (**b**), volume of the produced catholyte (**c**) and pH and conductivity values of the catholyte (**d**).

Figure 2a shows power performance of all tested MFCs, where six individual reactors were assembled, inoculated and operated in the same manner. Following inoculation, the MFCs were fed with neat human urine in continuous flow, at a flow rate of 10 mL/h reaching steady state after 15 days. After 12 days of operation, generation of catholyte liquid was observed in all MFCs and quantified as shown in Fig. 2c. The extracted catholyte was transparent in colour (Fig. 2b) and the rate of catholyte production was estimated to be between 0.35–0.65 mL/day (Fig. 2c). The pH ranged between 13.4 and 11.0 while the inlet urine pH was 9.2 (Fig. 2d). This is the highest pH of produced catholyte from the MFC operated on urine reported so far and it suggests a high caustic content as reported previously in ceramic MFCs operated on wastewater supplemented with acetate^21^.

The quantity and quality of the catholyte is dependent on MFC design, type of feedstock, power output as well as the type, porosity and thickness of the separator. It is suspected this is due to the lower porosity of the ceramic material used for this study compared to previously used terracotta of the same type and thickness^12^ where the electroosmotic movement is more prominent. Ceramic diaphragms or separators have been used in electrokinetic studies including electro-osmosis^26^ and it shows a movement of cationic species dragging water molecules from the microbial anode to the cathode^13,25^. This creates great opportunity for the MFC based recovery of nutrients, energy and water^27^, as well as ammonia stripping^18,21^ and it creates a self-hydration mechanism for the cathode mitigating biofouling and accumulation of salts on air-breathing cathode^16^. Although the volume of the produced catholyte is low, this line of research aimed to present the production of high quality catholyte for the first time in urine operated MFCs and it aims to further optimise the type of ceramic separator and the design to achieve higher production rates.

The catholyte conductivity values were between 27 and 57 mS/cm as shown in Fig. 2d which, is higher than urine used as the feedstock, which in turn might suggest ion concentration and accumulation within the cathodic half-cell as well as ion splitting induced by the electro-generation of current and kinetic movement of ions from the anodic to the cathodic half-cell. The relationship between pH and conductivity in Fig. 2d might suggest that these parameters in this case are interdependent.

### MFC performance under closed and open circuit

**Figure 3:**
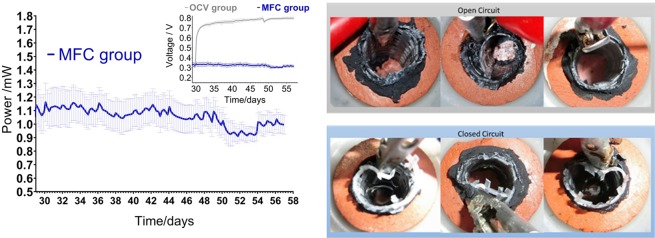
Power output from the MFC group, where the inset shows MFC voltage output under open circuit (OCV group) and closed circuit (MFC group) conditions. Cathode chambers after 7 days of operation where the catholyte is formed in closed circuit group and cathodes are empty in open circuit group.

**Figure 4:**
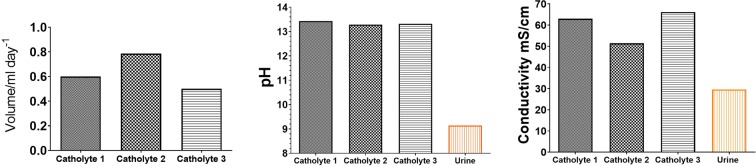
Catholyte properties collected from the MFC group.

After 30 days of MFC operation and reaching steady state, all six MFCs were divided into two groups to observe the catholyte production. The first group had the external load removed (OCV group) while the second (MFC group) was kept under closed circuit with an external load applied (100 Ω). After 7 days of operation as shown in Fig. 3, the voltage of the OCV group reached 750 mV while the MFC group maintained the steady state performance at ~334 mV which is equivalent to 1.1 mW of raw power production from a single MFC, and this is equal to a power density of: 15.3 Wm^−3^. The image in Fig. 3 illustrates the cathode chambers in the OCV group photographed after 7 days of operation and it showed no liquid presence in the cathode chambers, while in the closed-circuit MFC group the catholyte was observed and collected (Fig. 4). This is an indication of current driven transport through the ceramic from the anode to the cathode resulting in the formation of liquid filtrate. This is supported by previously reported electroosmotically driven extraction through MFC^28^ that also resulted in clear, transparent catholyte from potassium acetate or sodium acetate enriched wastewater^15,28^. However, in these previous studies, the additional osmotically driven transport passively moving liquid across the membrane was reported which can be quantified in open circuit mode of operation while in this work this process is completely excluded as the OCV reactors did not produce any catholyte. This again might be related to ceramic porosity as the chosen ceramic in this study is less porous (12.3% porosity measured by water absorption) than previously used terracotta chassis (14.5%)^12^.

During the tests, the rate of catholyte production was quantified once more and it showed up to 0.79 mL day^−1^ with pH values of 13.3–13.4 and conductivity ranging between 51.4 and 66.6 mScm^−1^. It shows up to 4.29 higher pH value than urine used as the feedstock and up to 33.6 mScm^−1^ higher conductivity value than urine (Fig. 4) suggesting that ionic concentration of the recovered catholyte is more than doubled compared with the initial urine feedstock. This significantly increased the concentration of catholyte via a MFC system has been reported previously using sodium or potassium acetate supplemented wastewater however it has not been reported for urine MFCs. This is the first time that highly alkaline solution of pH>13 was recovered from urine operated MFCs as a result of electric current production in ceramic bioreactors. The pH and conductivity values suggest this filtrate possesses antimicrobial and disinfecting properties. Catholyte samples from Fig. 4 were then used in the inactivation tests.

### Polarisation experiments

**Figure 5:**
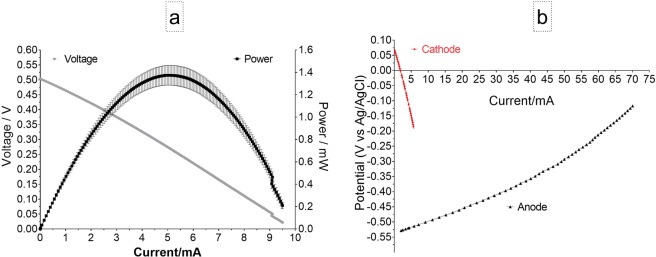
Polarisation and power curves showing of the performance of the MFC group (triplicate) (**a**), Linear Sweep Voltammetry of the anodes and cathodes in MFC group (**b**).

Polarisation curves in Fig. 5 indicate that the open circuit value was 505 mV and short circuit current was 9.6 mA while the maximum power point was 1.37 mW (mean value) and, which corresponds to a volumetric power density of 19.0 Wm^−3^. The LSV of the anode half-cells showed that the rate of voltage decreases when the current increases and that this rate is lower on the anode indicating that the cathode is the limiting factor.

#### Impact of neat and diluted catholyte on *Salmonella typhimurium*

Microbial fuel cell technology using ceramic membrane separator has made possible the synthesis of a unique solution with similar characteristics as concentrated sodium hydroxide. The characteristics of the catholyte indicate its suitability as a natural and sustainable bactericidal agent^21^.

**Figure 6:**
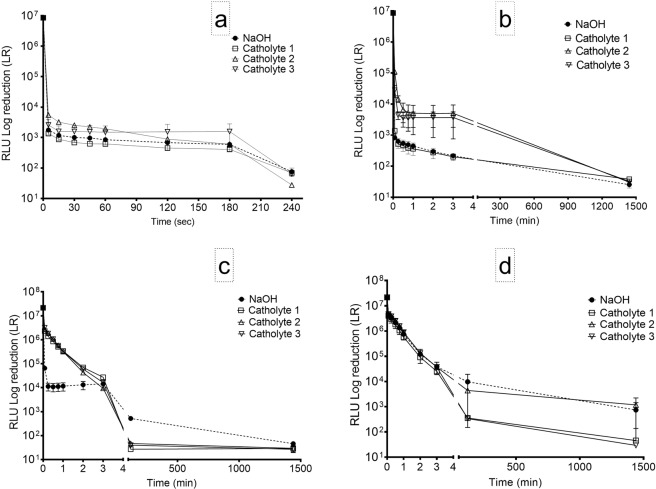
Pathogen kill rates of catholyte and NaOH used as control (**a**) Neat (**b**) 1:1 (**c**) 1:5 (**d**) 1:10 dilutions with deionised water (pH 6.9) using bioluminescent *Salmonella typhimurium*.

Figure 6a shows the results from the introduction of neat catholyte recovered from 3 different MFCs (pH ~ 13.4) as well as 1 M NaOH (pH 13.4) into a culture 10^8^ bioluminescent *Salmonella typhimurium*. A significant reduction in relative light units (RLU) was recorded in all tests when neat killing agents were applied to the organism (Fig. 6a). Catholyte from MFC 1 achieved the greatest reduction in the viability of the pathogen within the first 15 s.

A gradual reduction was recorded over 4 minutes resulting in the almost total killing of the pathogen with catholyte from MFC 3. At the end of the 4 minutes of RLU analysis, catholyte from MFC 3 produced more than 5 log-fold reduction in pathogen viability (Fig. 6a). At least 5 log-fold reduction was recorded from the catholytes from MFCs 1 and 2 as well as from the positive control (1 M NaOH), which indicates no significant difference in their killing efficacy at 4 min.

The result of the 1:1 dilution of the catholyte samples and sodium hydroxide showed differences in the killing rates of the diluted solutions. Addition of the diluted catholyte from MFC 1 and diluted sodium hydroxide to the pathogen resulted in more than 4 log-fold reduction within the first 60 sec, whereas just over 3 log-fold reduction was recorded from the catholyte of MFCs 2 and 3. Nevertheless, when given more reaction time (24 hr), more than 5 log-fold reduction was recorded in all diluted solutions (Fig. 6b). The result of 1:5 dilution revealed a more rapid killing of the pathogens by the diluted sodium hydroxide leading to more than 3 log-fold reduction in pathogen numbers within the first 60 s. There was however, a slower rate of killing recorded from the catholyte solutions. Unlike the sodium hydroxide, catholyte brought about 2 log-fold reduction in the first 60 s, however, by 3 min, the rates of killing recorded from the catholyte had matched the rate observed from the sodium hydroxide. Within one hour, the killing recorded for catholyte solutions had reached almost 6-log-fold reduction compared to just over 4 log-fold reduction recorded for sodium hydroxide solution. At the end of the 24 hr analysis, about 6 log-fold reduction was recorded in all solutions, including the sodium hydroxide (Fig. 6c). A consistent similarity was observed in the killing rates recorded for 1:5 dilution of all catholyte samples.

To further test the impact of dilution on the potency of the catholyte as a bactericidal agent, a further 1:10 dilution was carried out. This resulted in the decline of pH from the initial 13.4 to 11.5, 10.7, 10.5, 10.9 for sodium hydroxide, and catholyte from MFC 1, 2 and 3 respectively (Table 1). The result of the 1:10 dilution revealed a slower killing rate compared to the previous dilutions. Only about 3 log-fold reduction was achieved by all the killing agents at 3 min. At this stage, there was no difference in the killing rates of the catholyte or sodium hydroxide. However, after 60 minutes, catholyte from MFC 1 and 3 have produced almost 5 log-fold reduction in pathogen numbers compared to 3 and 3.5 log-fold reduction for sodium hydroxide and catholyte 2 respectively. After 24 h exposure of the pathogen to the diluted killing agents, catholyte originating from MFC 1 and 3 produced more than 6 log-fold reduction while just over 4 log-fold reduction was recorded from catholyte from MFC 2 and sodium hydroxide solutions (Fig. 1d).

**Table 1:** pH of the catholyte solutions and NaOH in neat and diluted form.

	Neat	1: 1	1: 5	1:10
**NaOH**	13.4	13.04	12.4	11.5
Catholyte 1	13.4	12.92	12.03	10.66
Catholyte 2	13.3	12.8	12.3	10.5
Catholyte 3	13.4	12.87	12.1	10.9

The strong correlation between bioluminescence and viability of microorganisms has made possible the evaluation of the effects of antimicrobial agents on bacteria in real time^21,29^. In the current study, we employed luminometry to evaluate the efficacy of catholyte as bactericidal agent. Analysis of the killing rates of the neat and diluted catholyte samples indicated that the synthesized catholyte was an efficacious bactericidal agent on known pathogenic bacteria, even when diluted up to 1:10. As expected, the rate of killing recorded was the highest in the neat sample (1.7 log reduction min^−1^) while 0.8-log reduction min^−1^ was recorded for catholyte solutions of 1:10 dilution. These results showed that dilution of catholyte has some impact on the potency. However, it is evident that with more reaction time, the diluted catholyte could achieve the same level of disinfection recorded as with the neat catholyte.

Gluhchev and Ignatov studied the impact of catholyte obtained from the electrochemical activation of aqueous solutions of sodium chloride on *E coli*^30^. The catholyte, which was made up of mainly NaOH, was found to be efficient in the suppression of the growth of *E coli*. Investigation by Swan *et al*.^31^ on the possibility of the use of catholyte (mainly NaOH) as a disinfectant to eradicate biofilm in hospital washbasin U-bend showed its efficacy. The results of their study showed that the catholyte was effective in eradicating bacteria biofilm present in the washbasin including *Pseudomonas aeruginosa*, *P. putida*, *Staphylococcus warneri, Staphylococcus epidermidis, Stenotrophomonas maltophilia*, and *Sphingomonas paucimobilis*. These results are consistent with finding in our study.

Previous studies involving the use of NaOH as a disinfectant found that the solution is effective against *Salmonella typhi* and *Staphylococcus aureus*, even up to 2% dilution in the presence of organic matter. However, a stronger concentration was recommended against spore–bearing organisms^32^. Complete inactivation of viruses, bacteria, yeast, fungi, prions and endo toxins has also been reported by the use of sodium hydroxide^33^. Catholyte synthesised from ceramic-based MFCs has been used in previous studies for the lysis and digestion of microalgae^34^ which might be an indication of its possible application in pre-treatment of biomass for energy generation.

#### Impact of neutralisation on the potency of the killing agents

To evaluate the contribution of pH to the killing potential of the killing agents, the catholyte and sodium hydroxide were neutralised with droplets of 1 M H_2_SO_4_ to pH 7.2 and 9.3. The resultant pH of the neutralised solutions is shown in Table 2. Formation of salt precipitates was observed in the neutralised catholyte samples.

**Table 2:** Resultant pH of the neutralised killing agents.

	Low pH	High pH
NaOH	7.3	9.3
Catholyte 1	7.2	9.27
Catholyte 2	7.34	9.33
Catholyte 3	7.2	9.26

**Figure 7:**
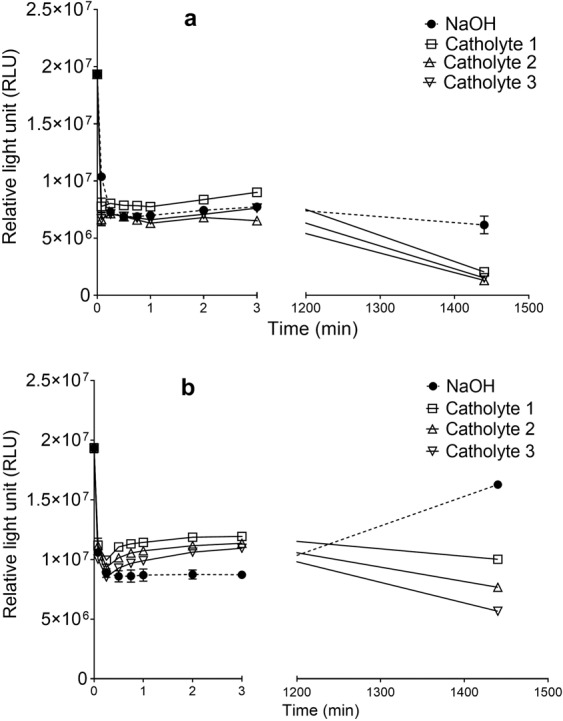
Pathogen killing rates of catholyte and NaOH neutralised to pH (**a**) 9.3 and (**b**) 7.2, using bioluminescent *Salmonella typhimurium*.

Results of the analysis of the neutralised killing agents showed that neutralisation weakened all the solutions, resulting in low killing rates (Fig. 7a,b). For instance, for solutions neutralised to pH ~7.3, 55% reduction in pathogen numbers was recorded after 3 min when sodium hydroxide was used compared to 38, 41 and 43% reduction recorded for catholyte solutions from MFCs 1, 2 and 3, respectively. Moreover, after 24 h, the results showed greater reduction in the neutralised catholyte of 48, 60 and 71% for MFCs 1, 2 and 3 respectively, compared to only 16% reduction from the neutralised sodium hydroxide (Fig. 7a). Results indicated recovery by the pathogenic organisms only in the solution containing neutralised sodium hydroxide. This result showed that the neutralised catholyte (pH 7.3), remain potent over a longer period compared to the neutralised sodium hydroxide solution, despite the similarity of their pH and the initial sluggish reactions. This is an indication that potentially, other reactive species are present in the catholyte responsible for the long-term potency of the catholyte solution.

Results of the killing rates recorded for neutralised solutions at pH ~ 9.3 revealed more potent killing generally when compared to the solutions at pH ~ 7.3. After 3 min of exposure of the pathogens to the killing agents, 60% reduction was recorded for the neutralised sodium hydroxide (pH 9.3) while the addition of the neutralised catholyte (pH ~ 9.3) from MFC 1, 2 and 3 resulted in 53, 66, and 61% reduction in pathogen viability. There was a similarity in the killing rates recorded from all killing agents at 3 min. However, after 24 h of exposure of the pathogens to the neutralised killing agents, only 68% pathogen reduction was recorded from sodium hydroxide while the neutralised catholyte from MFC 1, 2 and 3 produced 89, 93 and 92% reduction in pathogen viability, respectively (Fig. 7b). Generally, neutralisation of the killing agents with the resultant salt formation (Fig. S1, Supplementary Information) suggest that most of the cations and some of the anions, which confer bactericidal activity as the killing agents (catholyte only) have been taken out of the solution resulting in weaker solutions (Fig. S1). However, the neutralised catholyte solutions exhibited greater level of disinfection than the sodium hydroxide under the same neutralised conditions. Possible killing agents would include Reactive Oxygen Species^23^, reactive chlorine^35^, ammonia based compounds^36,37^ or other and it is a subject of further investigation. From the perspective of nutrient recovery, pH > 13 enables the volatilisation and recovery of nitrogen by the formation of ammonia^18^ and the content remaining in the liquid phase still needs to be studied.

**Figure 8:**
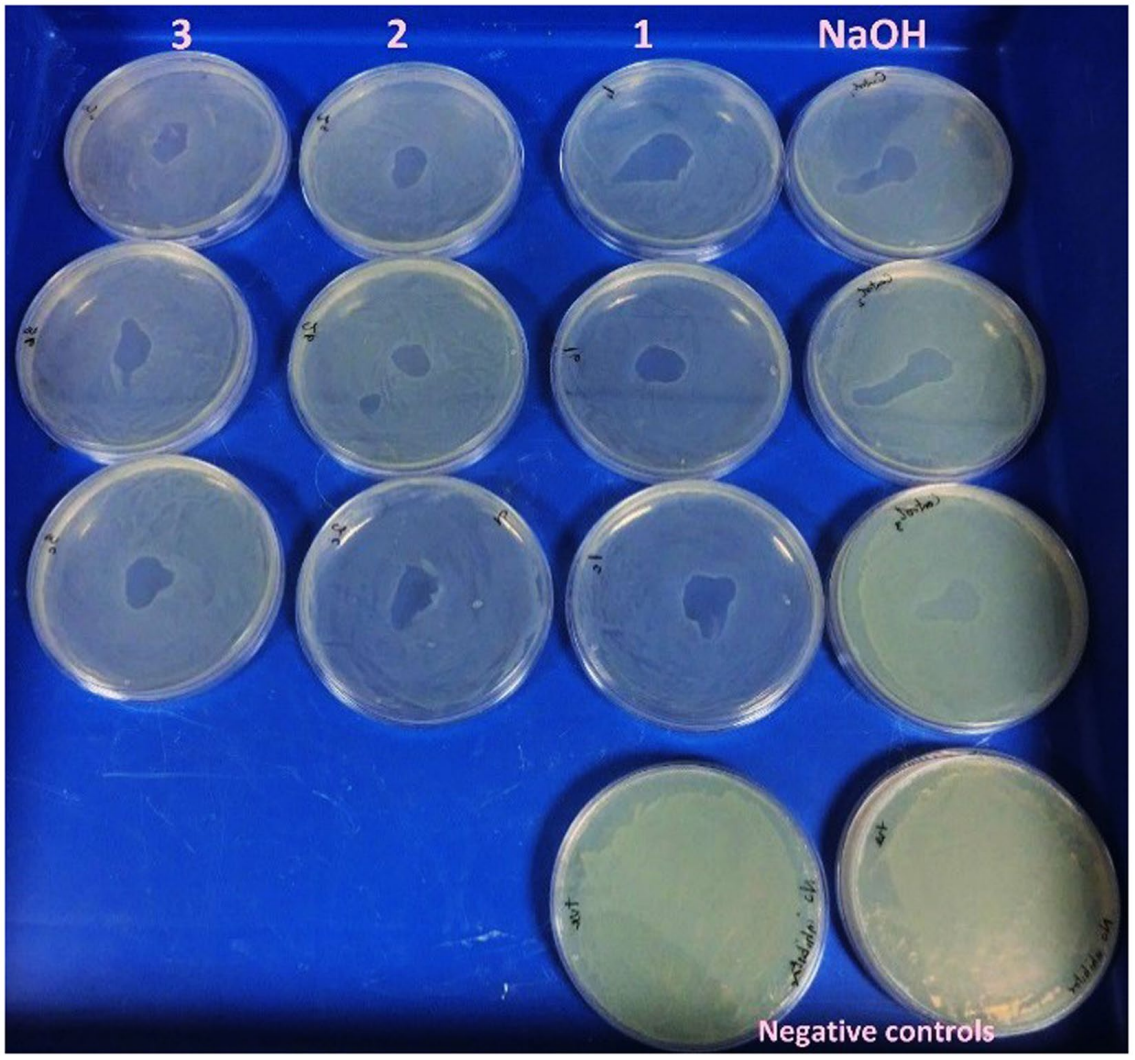
A droplet of the 50 μl of catholyte was applied in the centre of an agar plate containing *Salmonella typhimurium* (10^8^/mL) in samples 1–3 and the NaOH was used as a positive control. No inhibitor used in the negative control plates.

To evaluate the long-term efficacy of neat catholyte as a killing agent, *Salmonella typhimurium* (10^8^/mL) was plated on a selective medium. A droplet of 50 µL of the neat undiluted catholyte was placed at the centre of the agar plate and incubated at 37 ^o^C in 3 replicates. The results showed that the catholyte was able to stop the spread of the pathogen in areas containing the catholyte, even after 10 days of evaluation. The impact of the catholyte on the spread of the organisms was similar to that of concentrated 1 M NaOH. There is also a strong indication that the volatile compounds probably ammonia, from the catholyte inhibited the growth of the pathogen in areas not directly in contact with the catholyte, compared to the agar plates containing NaOH (positive control) and negative controls (Fig. 8).

Generally, the similarity recorded in the activities of concentrated NaOH and catholyte solutions in this study, suggests that the sustainably synthesised catholyte could be employed for some of the disinfecting applications where NaOH is normally used, without any damage to the environment. Both ionic and organic content of the catholyte needs to be studied further to explore the composition as well as the types of antimicrobial agents as appropriately designed MFCs have shown promise as an energy-efficient alternative for the recovery of useful compounds from urine.

## Conclusions

In this study, the microbial fuel cell operated on human urine was shown to produce clear transparent catholyte as a product of electricity generation through electroosmotic drag. Catholyte was then used *ex situ* as a killing agent to deactivate pathogen *Salmonella typhimurium* and proved to be a promising solution for water disinfection and purification. In the longer–term, the current production (i) alongside with the bioelectrocatalysis in the anode and waste being treated (ii), together with the electrically related treatment of waterborne pathogens (e.g., through disinfection or stimulated reactive oxygen species release) in the cathode (iii), represents a promising advantage of MFC systems. By this means, the extracted energy from waste does not have to be utilised within the reactor to further enhance the treatment efficiency but both processes are coexisting.

## Supplementary information

Supplementary Information.
