# Supplementary material for: Electroosmotically generated disinfectant from urine as a by-product of electricity in microbial fuel cell for the inactivation of pathogenic species
Source: Sci Rep. 2020 Mar 26;10:5533. doi: 10.1038/s41598-020-60626-x (PMC7099033; doi:10.1038/s41598-020-60626-x)
Supplement: Supplementary file 1 — Supplementary Information. [file 41598_2020_60626_MOESM1_ESM.docx]

**Supplementary Information**

**Electroosmotically generated disinfectant from urine as a by-product of electricity in Microbial Fuel Cell for the inactivation of pathogenic species**

Iwona Gajda^a,*^, Oluwatosin Obata^a^, John Greenman^a,b^, Ioannis A. Ieropoulos^a,b*^

^a^ Bristol BioEnergy Centre, Bristol Robotics Laboratory, University of the West of England, BS16 1QY, UK

^b^ Biological, Biomedical and Analytical Sciences, University of the West of England, BS16 1QY, UK

^*^Corresponding author: [Iwona.Gajda@uwe.ac.uk](mailto:Iwona.Gajda@uwe.ac.uk)


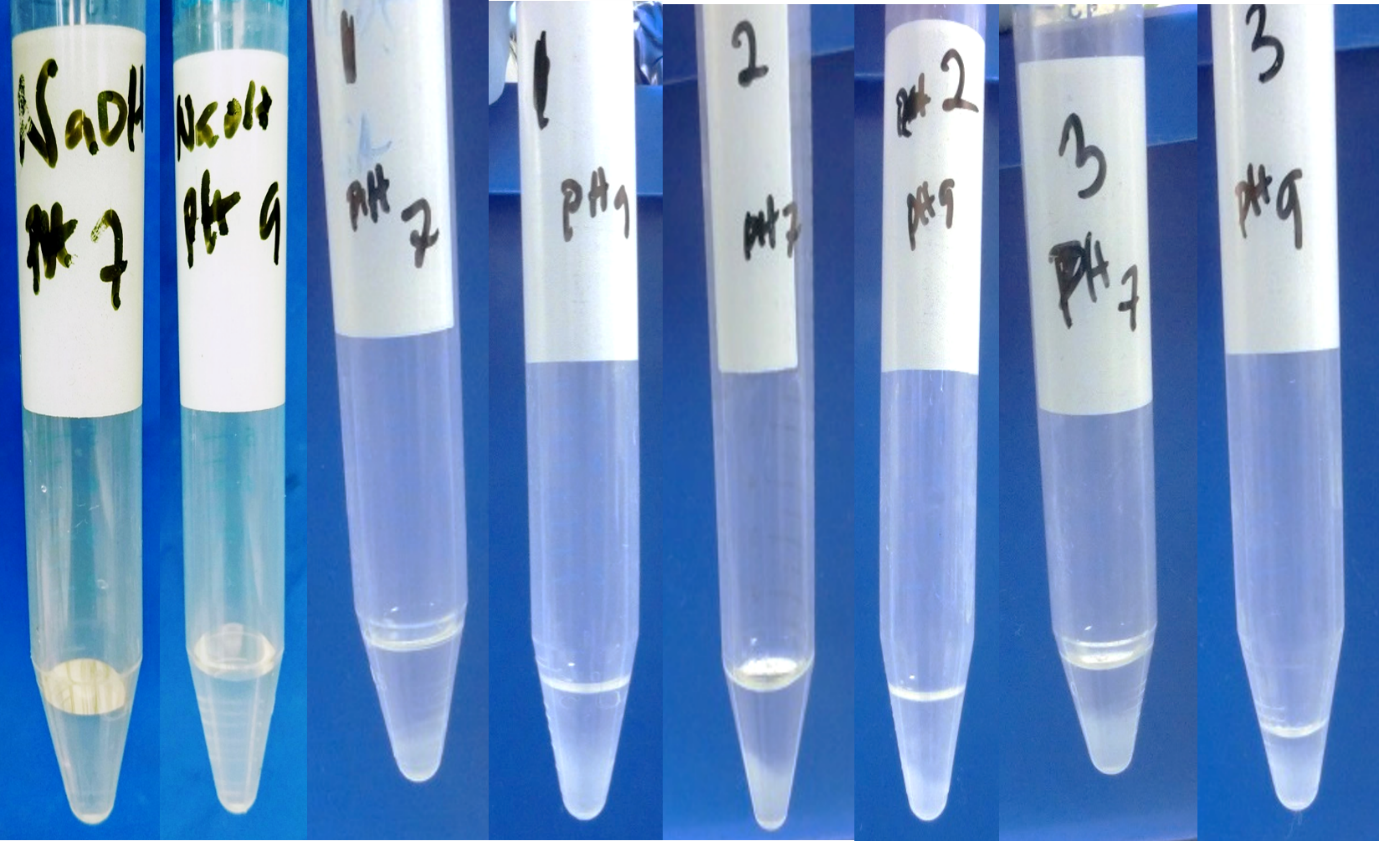


Fig S1. Images of killing agents after neutralization showing salt formation in the catholyte solutions.
